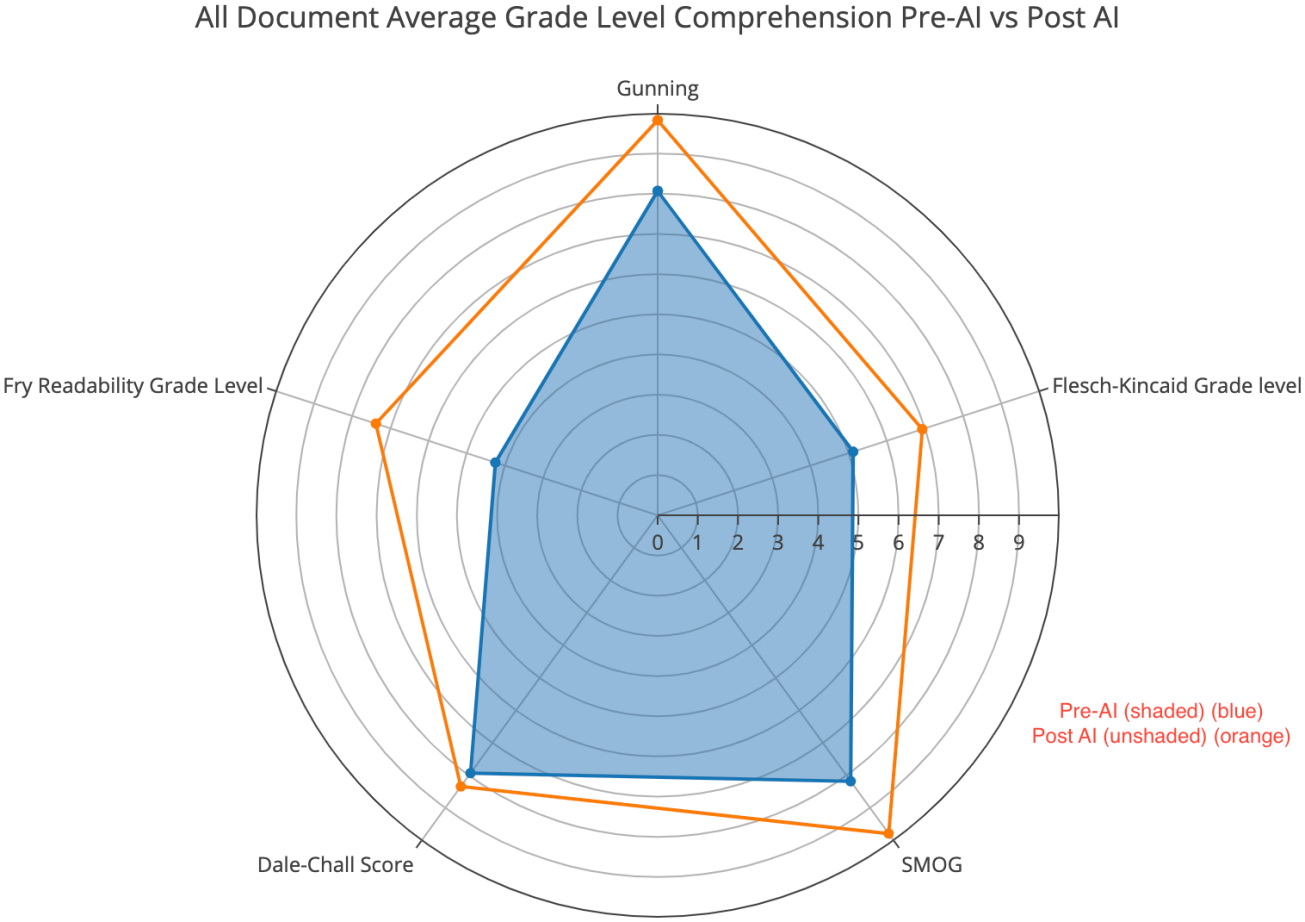# 658 Artificial Intelligence Does Not Increase Readability in Burn Education for Patients Without a Medical Background

**DOI:** 10.1093/jbcr/iraf019.287

**Published:** 2025-04-01

**Authors:** Vincent Athas, Muhammad Mustafa, Michelle Broers, Haily Smith, Elizabeth Smith, Matthew Bozeman

**Affiliations:** University of Louisville Burn Center; University of Louisville; University of Louisville Burn Center; University of Louisville; University of Louisville; University of Louisville

## Abstract

**Introduction:**

Patient education for burn victims in the post hospital setting remains a challenging endeavor. During the beginning of 2024 our Burn Center unveiled a bundle of short documents for distribution to burn patients in the outpatient setting pertaining to burn care. In our state individuals without a high school diploma remains 2% greater than the national average. As a next step in quality improvement, the educational materials were analyzed for several metrics approximating reading comprehension levels and then rewritten with the assistance of artificial intelligence (AI) software. Our hypothesis was that utilization of artificial intelligence would improve comprehension across all metrics allowing easier access to burn education for patients from all education backgrounds.

**Methods:**

Eight documents that underwent widespread distribution in the burn clinic at the start of 2024 were analyzed utilizing open-source readability analyzer. There were various original human authors. Utilizing open-source AI software the text was submitted with a command requesting simplification of the text. The results were reanalyzed for comprehensibility.

**Results:**

All documents initially written by human authors could be understood on average by patients with a 6th grade education (6.7). Open-source artificial intelligence software increased the comprehension difficulty across all metrics (Figure 1). Average percentage of polysyllabic words and long sentences increased to 69.3% from 62.0% with AI usage. Dale-Chall Score, a metric based on 3000 common words familiar to 4th graders, had the least amount of average change (8.14 to 9.3) compared to other metrics after AI modification.

**Conclusions:**

Clinical human authors can create documents for burn patients that are easily readable with only a grade school education. While AI may facilitate stylistic improvements, it appears that at least open-source software presently available only increases the education level required for comprehension.

**Applicability of Research to Practice:**

This process of comprehension analysis is easily reproducible with virtually no cost. With advances in the future in AI technology, any burn center can quickly apply a similar process to reach more patients from lower educational backgrounds.

**Funding for the Study:**

N/A